# Reconstructing dietary practices at Tell Kamid el-Loz (Lebanon) during the Bronze and Iron Age III / Persian to Hellenistic periods using plant micro-remains from dental calculus and stable isotope analysis of bone collagen

**DOI:** 10.1007/s12520-024-02000-w

**Published:** 2024-07-24

**Authors:** Shira Gur-Arieh, Stefanie Eisenmann, Amanda G. Henry, Mary Lucas, Daniela Lenz, Ptolemaios Paxinos, Hélène Weber, Lionello F. Morandi, Jeffery R. Stone, Michael Schultz, Patrick Roberts, Philipp W. Stockhammer

**Affiliations:** 1 Institute for Pre- and Protohistoric Archaeology and Archaeology of the Roman Provinces, Ludwig Maximilian University Munich, Munich, Germany; 2 Seminar for the Old Testament, Faculty of Theology, Humboldt University, Berlin, Germany; 3 Faculty of Archaeology, Leiden University, Leiden, The Netherlands; 4 isoTROPIC Research Group, Max Planck Institute of Geoanthropology, Jena, Germany; 5 The Arctic University Museum of Norway, UiT The Arctic University of Norway, Tromsø, Norway; 6 Institute for Palaeoanatomy, Domestication Research and History of the Veterinary Medicine, Ludwig Maximilian University, Munich, Germany; 7 Staatssammlung für Paläoanatomie, Staatliche Naturwissenschaftliche Sammlungen Bayerns, Munich, Germany; 8 Archaeometry Research Group, Eberhard-Karls-Universität Tübingen, Tübingen, Germany; 9 Department of Civilisations and Forms of Knowledge, University of Pisa, Pisa, Italy; 10 Department of Earth and Environmental Systems, Indiana State University, Terre Haute, IN USA; 11 Institute of Anatomy and Embryology, University Medical School Göttingen, Göttingen, Germany; 12 Institute of Biology and Chemistry, University of Hildesheim, Hildesheim, Germany; 13 Department of Archaeology, Max Planck Institute of Geoanthropology, Jena, Germany; 14 Max Planck Institute for Evolutionary Anthropology, Leipzig, Germany

**Keywords:** Kamid el-Loz, Middle Bronze Age, Iron Age III / Persian-Hellenistic, Diet, Dental calculus, Stable isotope analysis, Bone collagen, Levant

## Abstract

Tell Kamid el-Loz (Lebanon) was an important Bronze Age urban center that dominated one of the central crossroads of the Ancient Near East, connecting Egypt and the Levant with northern Mesopotamia, Anatolia, and Syria, as well as the interior with the Mediterranean coast. However, by the early Iron Age, the site had shrunk to a small rural settlement. Later, in the Iron Age III / Persian-Hellenistic, only enigmatic pits and a large cemetery remained. In this paper, we analyzed plant micro-remains from the dental calculus of 15 individuals (3 from the Middle Bronze Age II and 12 from the Iron Age III / Persian-Hellenistic) and *δ*^13^C and *δ*^15^N stable isotope data from tbulk bone collagen of 74 individuals (10 from the Middle Bronze Age II and 64 from the Iron Age III / Persian-Hellenistic) and 13 Late Bronze Age animal bones (7 *Ovis/Capra* and 6 *Bos*). Our results indicate general stability of human diet throughout the Middle Bronze Age II and the Iron III / Persian-Hellenistic periods, with a reliance on C_3_ plant crops and terrestrial animals also consuming C_3_ plants. In the later period, the plant micro-remains indicate the consumption of C_4_ plants and sedges, and the stable isotope analysis indicates differences in diet between males and females.

**Supplementary Information:**

The online version contains supplementary material available at 10.1007/s12520-024-02000-w.

## Introduction

Dietary practices of various Ancient Near East (ANE) populations have long been a focal point of research (e.g., Altmann and Fu 2014; Bottero 1985; Hastorf 2016; Samuel 1996). The importance of food for nutrition and survival, together with its pivotal role in cultural and self-defined identity, has led researchers to use dietary practices as indicators for long-distance trade (e.g., Weiss et al. 2019), ethnicity (e.g., Faust 2019; Mahler-Slasky and Kislev 2010; Sapir-Hen 2019), and status (e.g., Van Neer and Ervynck 2004) in a number of archaeological contexts. Given the important position of the Near East in discussions of the origins of agriculture (Zohary et al. 2012), the growth of proto-global exchange networks in general and across the Indian Ocean (Gilboa and Namdar 2015; Scott et al. 2021), the emergence of urbanism (Lawrence et al. 2022), and the experience of people under different imperial powers over the last three millennia (Tyson 2014), studies of dietary change, and its relevance for social, economic, and political organization, have been particularly intensive in this region (e.g., Chasan et al. 2022; Marom et al. 2009; Schmandt-Besserat et al. 2001).

Dietary change in the ANE has traditionally been studied through the examination of animal bones and charred plant remains (e.g., Frumin et al. 2023; Orendi and Deckers 2018; Vermeersch et al. 2021), although recent advances in interdisciplinary scientific methods allow greater insight into dietary patterns (Al-Bashaireh and Al-Muheisen 2011; Chowdhury et al. 2021; Langgut et al. 2016; Linares et al. 2019; Namdar et al. 2013; Sandias and Müldner 2015; Schutkowski and Ogden 2011; Scott et al. 2021; Sołtysiak and Fernandes 2021). While macro- and micro-botanical studies and stable isotope (SI) analysis of skeletal remains have become more common, multi-proxy analysis of both stable isotopes on bone collagen and plant micro-remains in Human Dental Calculus (HDC) from the ANE has not been carried out. Dental calculus is a unique reservoir that traps and preserves microparticles and chemical compounds ingested during an individual’s life (Radini et al. 2017) and is an invaluable source of information on plant utilization and consumption in prehistory (Henry et al. 2011).

The two most informative plant microfossils found in HDC are phytoliths (silicified plant cells or intercellular spaces) and starch grains, but additional microparticles including fungal spores, pollen, and diatoms can also be found. Starch grains and phytoliths have diagnostic morphologies enabling identification of plant taxa and anatomical origin. *δ*^13^C and *δ*^15^N SI analysis of bulk bone collagen (hereafter bone collagen) provides information on the consumption of C_3_ versus C_4_ plants as well as the contribution of different animal protein sources to the diet (Richards 2020). Although *δ*^13^C and *δ*^15^N analysis have been used to reconstruct diets of individuals and populations at various sites in the ANE (some examples from the Levant include Al-Bashaireh and Al-Muheisen 2011; Sandias and Müldner 2015; Schutkowski and Ogden 2011; Sołtysiak and Fernandes 2021), they have mostly been studied in isolation from other dietary proxies.

In this paper, we present a study of plant remains from HDC and SI from bone collagen from Middle Bronze Age II (MBA II) and Iron Age III / Persian to Hellenistic populations (IA III / Persian-Hellenistic) from Tell Kamid el-Loz, one of the major sites in the under-studied Central Levant (Table 1). Sitting on major crossroads connecting Egypt and the Southern Levant, coastal Syria, and the urban centers of northern Mesopotamia and Anatolia, urban Kamid el-Loz of the MBA II and Late Bronze Age (LBA) exerted control over its surroundings and activity passing through the area (Kuschke 1966; Heinz 2000). During the Iron Age I and II periods (IA I-II) the site's importance diminished, and by the Iron III / Persian-Hellenistic periods only a small rural settlement and cemetery remained (Heinz 2016).

**Table 1:** Simplified chronology of Tell Kamid el-Loz from the Middle Bronze Age onwards, including settlement history and associated burials. The burials analyzed for this study are marked in bold. (The data for this table was compiled from: Hachmann and Penner 1999; Heinz 2016; Miron 1982; Wagner-Durand 2014)

Period	Rough absolute dates	General characterization	Main features	Burials
MBA I	2000–1750 BCE	Pre-urban (?)	Buildings	
MBA II	1750–1550 BCE	City 1	Fortification, residential area on northern slope	2 domestic burials
		Destruction	Fire in the palace and temple area	
		First Anomie (hiatus)	Northern slope abandoned, scattered activity in temple area	**Burial ground northern slope**, empty grave in temple area
		City 2	Fortification, MBA II Palace (MBP 2) and administrative area, Temple T5 + T4 Residential areas (north and west)	
		Destruction	Fire in palace, administrative area, and residential area in north	Mass grave Gt 1–3?
		Second Anomie (hiatus)	‘Intermediate building’ on top of the palace (MBP 1), traces of activities in the administrative area and temple ruins	GP1 (child grave) Mass grave Gt 1–3?
LBA I	1550/1450–1400 BCE	City 3	Palace (P5, P4, P3, P2/1), workshop area, temple (T3, T2, T1), residential areas	Child graves Gt 5–7: either late MB or LB; Treasury (end LB I to beginning LB II); LB II: burial ground 3 km away
LBA II	1450/1400–1200/1150 BCE			
IA I	1200/1150–1000 BCE	Village	‘Flimsy‘ remains of buildings	
IA II	1000–538 BCE	Decline in settlement activities	‘Flimsy‘ remains of buildings	
IA III / Persian	538–330 BCE	No settlement (?) only graves	Pits	IA III / Persian-Hellenistic Cemetery: **NW cemetery**, NE group, E cemetery
Hellenistic	330–64/30 BCE	Settlement	Houses IV and V	
Roman	From 64/30 BCE on	Settlement	House I-III	3 sarcophagi
	first century CE	Decrease in settlement activities		
Late Antiquity to modern era		Some settlement activity		Grave 73 (Late Roman-Byzantine)
				Cemetery (hundreds of graves, no grave goods)
Present			Mosque	Cemetery in southeast

Given the period and region investigated, we anticipated finding evidence for the consumption of predominantly domesticated plants and animals. However, considering changing settlement patterns and the diachronic gap between the burials investigated, we hypothesized there would be notable changes in dietary practices over time. This multi-proxy study revealed both long-term continuity in plant and animal consumption, as well as revealed diets that differed according to age and sex. Importantly, this study currently provides our only direct insight into dietary practices in the Central Levant during the IA III / Persian-Hellenistic periods.

### Tell Kamid el-Loz

**Fig. 1:**
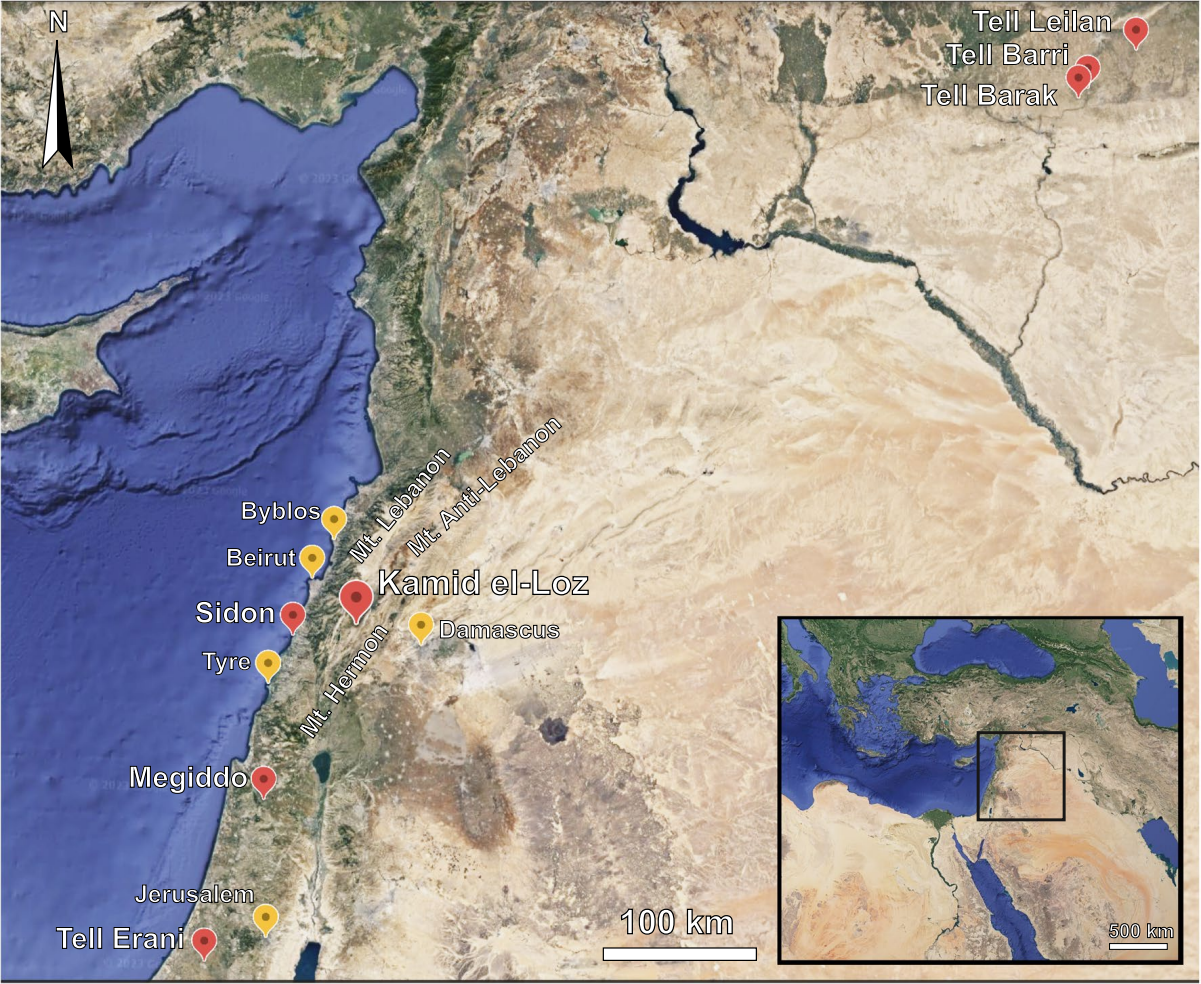
Map of the Central and Southern Levant. (Sites in red are mentioned in the text; sites in yellow are given as general reference. (Map modified by author from Google Earth)

Tell Kamid el-Loz (33°37′25"N 35°49′16"E, ~ 950 m above sea level) is located on the south-eastern edge of the Beqa’a Valley (Lebanon), where the alluvial plains meet the Aarbi mountain foothills (Fig. 1) (Heinz et al. 2001; Sommer 2001). The tell rises 26 m above the valley floor, providing a commanding view of the valley. The area is characterized by a Mediterranean climate with hot and dry summers and cold and rainy winters averaging ~ 550 mm of precipitation per year (Central Administration of Statistics 2010). The site is located at the edge of the Eumediterranean vegetation belt (800–1000 m) that is characterized by degraded *Quercus calliprinos* forests accompanied by other trees and shrubs such as *Sarcopoterium spinosum* (Hajar et al. 2010)*.* A perennial spring is located just north of the tell, and today flows into a dammed lake. Another spring that supplies water only during the winter months is located at the western edge of the tell (Hachmann 1989; Heinz 2016).

Kamid el-Loz was first excavated from 1963–1965 by Johannes Gutenberg University Mainz and Saarland University under the direction of Arnulf Kuschke and Rolf Hachmann. Excavations continued under the sole direction of Hachmann between 1966–1981, followed by renewed excavations by Freiburg University under Marlies Heinz from 1995–2011 (Hachmann and Kuschke 1966; Hachmann 1982, 1989, 1998; Heinz 2010, 2016; Poppa 1978). The projects revealed a multilayered tell with evidence of human occupation from the Early Bronze Age IV (EBA IV)/Middle Bronze Age I (MBA I) transition around 2000 BCE until today (Heinz 2016).

**Fig. 2:**
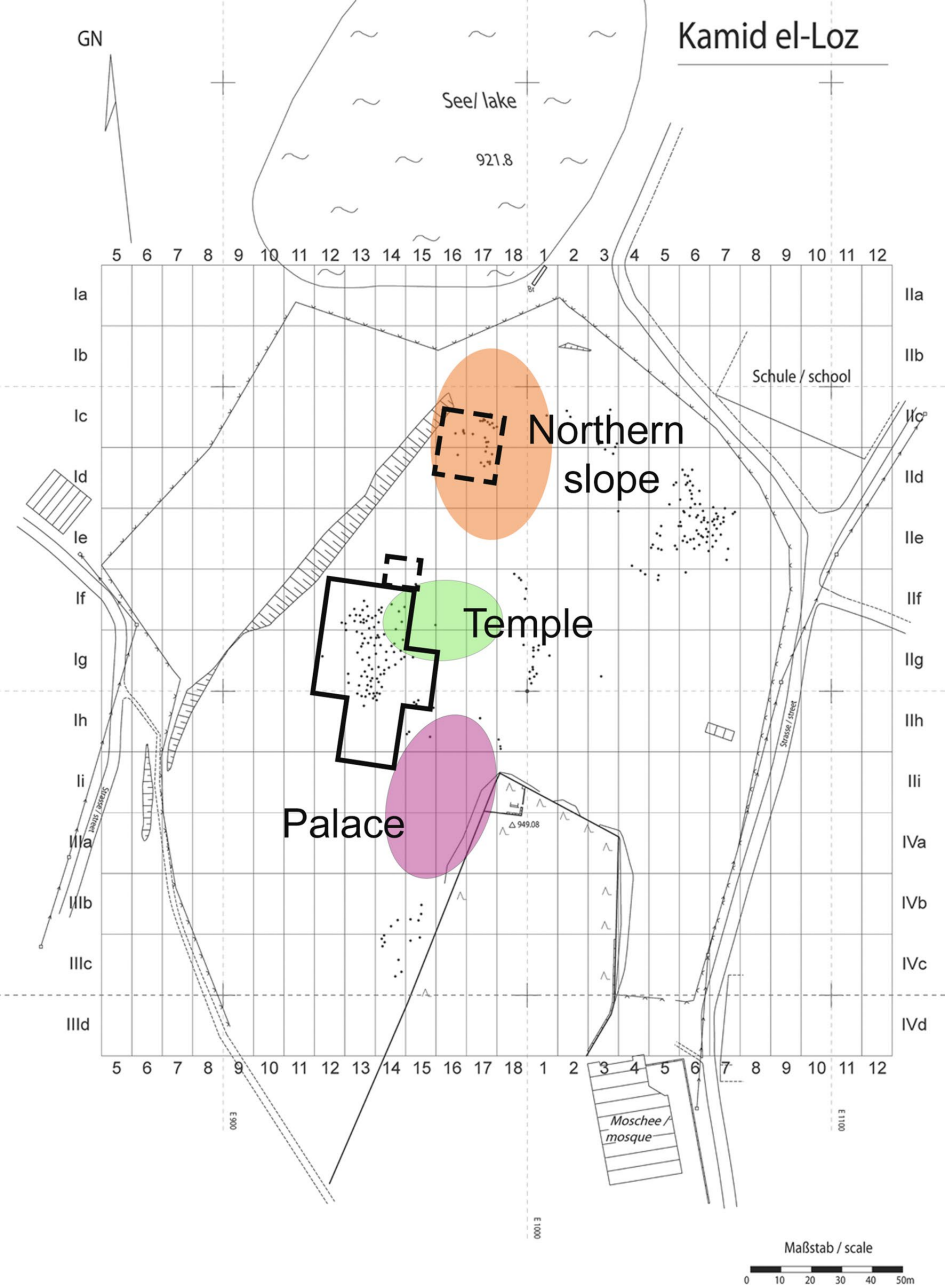
Plan of Kamid el-Loz with grid-system (excavations by M. Heinz, University of Freiburg, 1997–2011) and grave locations marked by black dots. The location of the relevant individuals from the Hachmann excavations studied in this paper are outlined in solid black (IA III / Persian-Hellenistic) and dotted line (MBA II burials). Note that the grid-system used by Hachmann from 1963–1981 was rotated to the east in comparison to the one presently in use. The colored circles indicate the location of important features. The plan shown here is modified from Wagner-Durand (2014: Fig. 1)

During the MBA II (1750–1550 BCE), the first urban settlement (City 1) developed including a large fortification system, temple, palace, administrative center, and residential areas on the northern slope. During this period, burials at Kamid el-Loz are rare with only two simple pit burials found in the northern residential area, one of an adult (G_113) and the other of an infant (G_96) (Miron 1982; Wagner-Durand 2014). The MBA II city was abandoned twice (‘First and Second Anomie’) following destruction by fire of unknown origin. Despite the first abandonment people continued to bury their dead at Kamid el-Loz as evidenced by seven adult and 18 child pit burials found in the ruins of the residential area in the northern slope (Miron 1982; Wagner-Durand 2014) (Fig. 2). After the first hiatus, a second MBA II urban settlement (City 2) was erected in the same place. (Hachmann 1989; Heinz 2016; Heinz et al. 2010, 2011; Metzger 2012). After the destruction of City 2 by a violent conflagration, traces of domestic and other activities were apparent in the administrative area and temple, and approximately 10 individuals were buried in the western residential area (Heinz 2016).

The third urban episode at Tell Kamid el-Loz (City 3) developed during the LBA I-II (ca. 1550/1450–1200/1150 BCE) and is identified as ancient Kumidi which is mentioned in the Amarna Letters (EA 116, 129, 132, 197, 198) found in the archive of Amenhotep IV (Akhenaten, 1353–1336 BCE) (Moran 1992). At this time, along with the Southern Levant, Kamid el-Loz was under the control of the New Kingdom of Egypt, possibly functioning as the seat of an Egyptian administrator during the fourteenth century BCE (Edzard 1970; Heinz 2010; Moran 1992; Wilhelm 1973). City 3 declined toward the end of the LBA, and during the IA I-II (1200/1150–538 BCE) the importance of the site decreased.

The IA I-II period remains are modest and rural without any monumental architecture. No burials from this period have been found so far (Heinz 2016). In the following IAIII / Persian period (538–330 BCE), the only evidence of human activity comes from large pits with unknown purpose and burials. During this period, large parts of the tell were used as a cemetery and hundreds of burials have been excavated to date, mainly from two areas (west and east) in the northern part of the site (Wagner-Durand 2014; Poppa 1978; Hachmann and Penner 1999) (Fig. 2). The burials continued into the Hellenistic period when people also returned to reside on the tell as evidenced by at least two large houses (Wagner-Durand 2014). Human activities on the tell continue into the present, with the mosque and cemetery of the modern Kamid el-Loz village located in the southeast part of the tell (Wagner-Durand 2014).

We hypothesized that general changes in settlement patterns, together with increased globalization in the Near East during the IA III / Persian-Hellenistic Periods (Geller 2014; Lehmann 2014), could have led to dietary changes at Kamid el-Loz. To test this hypothesis, we conducted a multi-proxy analysis to reconstruct the diet of individuals from the MBA II and the IA III / Persian-Hellenistic periods studying both plant micro-remains from dental calculus and stable isotope analysis of bone collagen. Importantly, these two methods provide direct and complementary information on individual diets. For example, while plant micro-remains provide high-resolution information on plant taxa introduced into the mouth environment, there is ambiguity regarding what mechanisms control their incorporation into HDC (Bartholdy and Henry 2022). Thus, it is not clear what time span microremains represent (e.g., weeks, years, or decades), or how microremains might reflect the broader diets of whole populations. In contrast, the SI analysis provides us with direct long-term insights on both plant and animal protein intake, albeit with low resolution regarding specific foods consumed. Together, the relative strengths of these two methods can provide a more comprehensive picture of dietary practices at Tell Kamid el-Loz over time.

### The Levantine diet and archaeological information on past dietary practices at Tell Kamid el-Loz

From the Bronze Age onwards, the subsistence practices of complex societies across much of the ANE were based on agriculture, horticulture, and animal husbandry. While recent studies using scientific methodologies demonstrate the diversity of plant food consumed in the ANE (see Fries-Knoblach and Stockhammer 2022 and references therein), the major crops found in archaeological sites are barley (*Hordeum vulgare* L.), emmer wheat (*Triticum dicoccum* Schrank), free‐threshing wheat (*Triticum aestivum/durum*), lentil (*Lens culinaris* Medik.), bitter vetch (*Vicia ervilia* [L] Willd), broad bean (*Vicia faba* L.), garden pea (*Pisum sativum* L.), and chickpea (*Cicer arietinum* L.) (Riehl 2014). Fruits such as olive (*Olea europaea* L.), grape (*Vitis vinifera* L.), fig (*Ficus carica* L. and *Ficus sycomorus* L.), pomegranate (*Punica granatum* L.), date (*Phoenix dactylifera* L.), and almond (*Amygdalus communis*) were also cultivated (Fries-Knoblach and Stockhammer 2022; Kamlah and Riehl 2020; Zohary et al. 2012). Vegetables are not often preserved in the archaeological record but are known to have been consumed from Mesopotamian textual sources and Egyptian tomb paintings and depictions dating to the Bronze Age. Watermelons (*Citrullus lanatus*), melons (*Cucumis melo*), aromatic plants from the genus *Allium* (onion, garlic, leek), beet (*Beta vulgaris*), and lettuce (*Lactuca sativaare*) are several of the vegetables consumed known from such sources (Zohary et al. 2012). Although somewhat overlooked in archaeology, wild plant seeds were also found at archaeological sites (e.g., Vermeersch et al. 2021), and no doubt some of them were consumed (e.g., Mahler-Slasky and Kislev 2010), as is still the case in some areas today (e.g., Baydoun et al. 2015; Marouf et al. 2015). Broomcorn millet (*Panicum miliaceum* L.) and foxtail millet (*Setaria italica*), both C4 plants unlike all the above-mentioned plants, appeared in the Near East relatively late during the MBA and became more common only in the IA as an important summer crop (Laugier et al. 2022; Riehl and Nesbitt 2003).

Sheep (*Ovis aries* L.) and goats (*Capra hircus* L.), as well as cattle (*Bos taurus* L.) commonly predominate ANE meat consumption (e.g., Kamlah and Riehl 2020; Lev-Tov and Whitcher Kansa 2017; Price et al. 2017). All three animal taxa were important not only for their meat but also for their secondary products such as wool and milk (Greenfield 2010; Sherratt 1981). Pig (*Sus scrofa domestica* L.) consumption was not uniform acrossthe various regions of ANE, although data suggest that pigs were of minor importance in the Southern Levant during the Bronze Age (Kamlah and Riehl 2020). In Northern Mesopotamia, at least during the EBA and MBA, pigs represented in some cases more than 50% of all identified bones at sites (Price et al. 2017). The occasional increase in pig consumption in the Iron Age, along with dog (*Canis familiaris* L.) consumption is commonly associated with the arrival of the Philistines to the Southern Levant, although this topic is highly debated (Kamlah and Riehl 2020; Maher 2013). Wild game animal bones are also found in many assemblages, indicating their enduring contribution to the diet despite their decreased importance over the Bronze and Iron Ages (Horwitz et al. 1999; Munro et al. 2018). Among the consumed game animals, the most common were gazelle (*Gazella* spp.), deer (*Dama dama* L.) wild boar (*Sus scrofa* L.), and hare (*Lepus* spp.) (Sapir-Hen 2022; Vermeersch et al. 2021). Various types of marine or freshwater fish were also consumed (Lernau et al. 2021; Van Neer and Ervynck 2004).

The archaeobotanical information from the past excavations at Tell Kamid el-Loz is very limited. Behre (1970) published six macro-botanical samples from LBA and Early Iron Age contexts, of which he only mentioned the edible plants. Barley (*Hordeum vulgare* L.) was the most common, appearing in all 6 samples, while dwarf wheat (*Triticum compactum*) and emmer wheat (*Triticum dicoccum*) were considerably less abundant. Of legumes chickpea (*Cicer arietinum*) and bitter vetch (*Vicia ervilia*) were the most abundant, followed by sweet pea (*Lathyrus sativus*). Lentils (*Lens culinaris*) and grapes (*Vitis vinifera* cf.) were represented by few isolated finds. A jar attributed to the LBA and filled with about 10 L of Viper's-bugloss seeds (either *Echium italicum* or *Echium glomeratum*) was published by Baas (1980) who suggested that the plant could have been used for medicinal properties, as a dye, or even as food. The limited published archaeobotanical information from Kamid el-Loz does not allow us to draw any conclusions, besides the fact that all the identified taxa are C_3_ plants common in Bronze and Iron Age Near Eastern sites. Vermeersch et al. (2021) studied the developments in subsistence practices from the EB I through the IA II (3600–586 BCE) in the Central and Southern Levant and identified differences between EB/MBA and LBA/IA plant assemblages. The earlier periods are characterized by the prevalence of emmer wheat and the later periods are characterized by large amounts of free-threshing wheat, flax, and pomegranate. Unfortunately for this study, the resolution of plant micro-remain analysis is not high enough to discern such differences.

Unlike the botanical remains, the faunal remains received more attention, culminating in a comprehensive monograph by S. Bökönyi (Bökönyi 1990). The following results are derived from his work. A sum of 13,301 bones were identified by Bökönyi at least down to genus level, the vast majority originating from LBA (n = 10,232 (~ 77%)) and Iron Age layers (n = 2401 (17.8%)). Only a small amount of the bones (n = 573 (4.45%)) were attributed to the MBA, possibly due to the relatively small number of loci of this period excavated by the end of the project in 1981 (Bökönyi 1990). Despite the relatively low number of identified specimens (NISP), the MBA sample is statistically significant given the total NISP exceeds 400. This number has been found to give a good representation of the relative importance of taxa in a sample, at least for the major domesticates (Paxinos 2017). Therefore, we can safely assume that sheep, and to a lesser extent goats, were already the most common domesticates in the MBA. Cattle bones, although fewer in number, are also common. Pigs and horses were also present albeit in low numbers. During the LBA, the importance of the small ruminant sheep and goats remains unchanged, although the ratio between them becomes more balanced, whereas the relative amount of cattle bone doubles. Interestingly, the absolute number of dog bones remains almost constant, and pigs remain present in low numbers. From the LBA onwards donkeys are attested for the first time, outnumbering horses, and can be regarded as one of the main changes during the Iron Age (Bökönyi 1990).

In all of the faunal assemblages studied by Bökönyi, wild animals did not play a substantial role in subsistence and he suggested that they were mostly hunted as a leisure activity of the elite (Bökönyi 1990, 1993). Judging from the habitat the identified wild animals encompass today, it is possible to reconstruct past environments, at least to some extent (Reitz and Wing 2008). The inhabitants of Tell Kamid el-Loz utilized resources from diverse areas such as woodlands (e.g., red deer, aurochs, wild boar, and brown bear), open environments (e.g., hare, onager), rocky terrain (e.g., bezoar goat), and lakes, rivers, and swamps (e.g., mallard, white-fronted goose). In addition, marine fish bones were also found at the site, probably transported from the Mediterranean coast (Bökönyi 1990). The diverse habitats attested by wild animal remains at Kamid el-Loz suggest that the area around the site was more forested in the past than in the present day (Bökönyi 1990), which is also supported by pollen data (Hajar et al. 2008).

## Materials and methods

The individuals studied here (Online Resource 1) were excavated in the 1960s and 1970s as part of the Hachmann excavations at Tell Kamid el-Loz (Miron 1982; Poppa 1978; Wagner-Durand 2014). The skeletal remains were exported in the’60 s and ‘70 s with the permission of the Lebanese Directorate of Antiquities and were first studied at the Department of Anthropology at Gießen University. They are currently curated in the anthropological collection of the Zentrum für Anatomie of the University of Göttingen. Of the 121 burials excavated, 15 individuals had enough dental calculus for analysis (three from the MBA II and 12 from the IA III / Persian-Hellenistic respectively). For full archaeological context and sampling information see Table 2. Of the 121 burials, 94 individuals produced rib bones with enough collagen for SI analysis, 12 from the MBA II and 82 from the IA III / Persian-Hellenistic (Online Resource 2).

**Table 2:** Information for the 15 individuals with HDC, including SI analysis results, and archaeological information. Skeletal sex and age were estimated by M. Kunter, following the methods outlined in 1977 (Kunter 1977). ND = not determined, Cyper = Cyperaceae, D = Diaton, MC = Micro-charcoal. The SI results for the other individuals are included in Online Resources 2 and 4. Tooth codes follow the FDI World Dental Federation notation (ISO 3950)

Grave no	Grave ID during excavation	Control-List number	Estimated age at death	Age group	Estimated sex	Grave goods	Relative date	Teeth sampled for HDC	Sample weight (mg)	*δ*^15^N (‰)	*δ*^13^C (‰)	Starch	Phytolith	Other
G_97	ID15: 3	KL 67:411a	> 60	Elder adult	F	High	MBA II	26, 37	ND	7.0	–19.4	T1 (TASH)		D, MC
G_99	ID15: 11	KL 68: 154 a-c	30–50	Adult	M	Low	MBA II	47, 48	ND	7.4	–18.8			D, MC
G_100	ID15: 14	KL 68: 184 a-g	40–50	Mature adult	F?	Medium	MBA II	33	ND	7.6	–18.8		(Few)	MC
G_3	IG13:8	KL 64:294	> 60	Elder adult	F	Medium	IA III/ Persian- Hellenistic	43	2	8.0	–19.4		(Few)	D, MC
G_15	IH13:15	KL 67:393, KL 68:3	30–40	Adult	F	High	IA III/ Persian- Hellenistic	47	6.3	7.4	–19.2	T2 (USO)	Sedge	MC
G_19	IH13:12	KL 68:218	20–25	Adult	F	Low	IA III/ Persian- Hellenistic	43	3.2	7.6	–18.9		Sedge, Panicoid	D, MC
G_39	IG12:9	KL 64:89	30–40	Adult	M	Medium/high	IA III/ Persian- Hellenistic	37	2.5	8.7	–19.4		Only Pooid	D, MC
G_48	IG12:20	KL 66:7, KL 66:31	30–40	Adult	F	NA	IA III/ Persian- Hellenistic	37, 38, 41, 42	2.1	7.8	–19.4	+		D, NPP (Alternaria sp.)
G_60	IH12:26	KL 66:376	40–50	Elder adult	F	High	IA III/ Persian- Hellenistic	42, 43	2.2	7.2	–20.1			D, MC
G_65	IH12:5	KL 66:88	30–35	Adult	F	Low	IA III/ Persian- Hellenistic	43, 46, 47	1.7	7.8	–20.2		Only Pooid	D, MC
G_66	IH12:13	KL 66:83	30–40	Adult	F	NA	IA III/ Persian- Hellenistic	36, 37	4.3	6.6	–18.9	+	Sedge, Panicoid	D, MC
G_67	IH12:12	KL 66:90	40–60	Mature adult	M	High	IA III/ Persian- Hellenistic	33	2.3	8.0	–18.8		Sedge	D, MC
G_71	IJ12:1	KL 68:254; KL 70:73	20–30	Adult	F	High	IA III/ Persian- Hellenistic	41, 42	2.1	7.2	–20.1	+		D, MC
G_86	IH12:24b	KL 66:632b	40–60	Mature adult	M	Medium/high	IA III/ Persian- Hellenistic	44	2.1	ND	ND		Sedge	D, MC
G_IIE 7:1	IIE 7:1	KL 64:339	30–40	Adult	M	NA	Likely IA III/ Persian- Hellenistic	47	2.1	ND	ND	T1 (TASH), T3 (AP)	(Few)	D

Individuals from the different cemeteries were divided into age groups based on osteoarchaeological assignments from the literature (Kunter 1977; Miron 1982). We simplified Poppa's (1978) division of the burials to infant (0–2.5 yrs), child (5–13 yrs), juvenile (14–19 yrs), and adult. In the adult category, we combined the adults (20–40 yrs), mature adults (40–60 yrs), and elder adults (> 60 yrs) as initial SI analysis pointed to no differences between them (Table 3). Biological sex was based on its assignment in the original publications (Kunter 1977; Miron 1982).

**Table 3:** Summary of the stable isotope results of different groupings. Two subsets were compared using Wilcoxon and more than two subsets using Kruskal–Wallis. The mean results are given with ± 1 standard deviation. The age categories are: infants (0.6–2.5 yrs), child (3–13 yrs), juvenile (14–19 yrs), adult (20–60 + yrs). Three IA III / Persian-Hellenistic individuals were not included in the age categories (two fetuses, G_35 and G_64, and one individual too disturbed to determine age at death (G_93)

	n	Min. *δ*^13^C (‰)	Max. *δ*^13^C (‰)	Mean *δ*^13^C and SD (‰)	Median *δ*^13^C (‰)	p-value* δ*^13^C (‰)	Min *δ*^15^N (‰)	Max *δ*^15^N (‰)	Mean *δ*^15^N and SD (‰)	Median* δ*^15^N (‰)	p-value* δ*^15^N (‰)
Animals											
LBA *Ovis/Capra*	7	–20.6	–19.5	–20.0 ± 0.4	–20.1		4.9	6	5.4 ± 0.4	5.3	
LBA *Bos*	6	–20.0	–18.4	–19.4 ± 0.6	–19.5		4.3	6.9	5.7 ± 1.1	5.8	
Total By Period Adults											
Total MBA II Adults	5	–19.8	–18.8	–19.2 ± 0.4	–19.2	0.6049	7.0	8.1	7.4 ± 0.5	7.4	0.0217
Total IA III/Persian-Hellenistic Adults	45	–20.5	–18.7	–19.4 ± 0.5	–19.2		6.6	10.4	8.3 ± 0.9	8	
MBA II Period											
MBA II Infants	2	–18.5	–18.0	–18.3 ± 0.4	–18.3		9.0	10.5	9.7 ± 1.1	9.7	
MBA II Children	3	–19.0	–18.8	–18.8 ± 0.1	–18.8		8.1	8.3	8.2 ± 0.1	8.2	
MBA II Adults	5	–19.8	–18.8	–19.2 ± 0.4	–19.2		7.0	8.1	7.4 ± 0.5	7.4	
IA III/Persian-Hellenistic Period											
IA III/Persian-Hellenistic Infants	5	–19.1	–16.5	–18.1 ± 1.1	–18.5	0.0013	9.3	11.6	10.5 ± 0.9	10.4	0.0006
IA III/Persian-Hellenistic Children	4	–19.9	–19.2	–19.4 ± 0.3	–19.4		7	8.9	7.5 ± 0.9	7.1	
IA III/Persian-Hellenistic Juveniles	7	–21.0	–19.5	–19.8 ± 0.6	–19.6		5.2	8.9	7.3 ± 1.2	7.4	
IA III/Persian-Hellenistic Adults	45	–20.5	–18.7	–19.4 ± 0.5	–19.2		6.6	10.4	8.3 ± 0.9	8	
Sex											
IA III/Persian-Hellenistic Female	20	–20.5	–18.9	–19.5 ± 0.5	–19.4	0.0116	6.6	9	7.8 ± 0.6	7.8	0.0275
IA III/Persian-Hellenistic Male	25	–20.5	–18.7	–19.2 ± 0.4	–19.1		7.1	10.4	8.5 ± 1.0	8.2	
Grave Goods											
MBA II low	2	–19.8	–18.8	–19.3 ± 0.6	NA		7.6	8.7	7.8 ± 0.3	NA	
MBA II medium	1	–19.4	NA	NA	NA		7	NA	NA	NA	
MBA II high	1	–18.8	NA	NA	NA		7.4	NA	NA	NA	
IA III/Persian-Hellenistic low	7	–20.5	–18.7	–19.3 ± 0.7	–19	0.3739	7.6	10	8.7 ± 1.1	8.2	0.771
IA III/Persian-Hellenistic medium/high	4	–19.7	–19.4	–19.4 ± 0.5	–19.5		7.6	8.7	8.2 ± 0.9	8.2	
IA III/Persian-Hellenistic high	21	–20.5	–18.7	–19.4 ± 0.5	–19.2		7.2	10.3	8.2 ± 0.9	8	

Unfortunately, nearly all the animal bones from the Hachmann excavation were lost, except for 17 baskets from the LBA kept as special finds. Archaeozoological analysis of this limited assemblage identified sheep and goat specimens as by far the most abundant species (Paxinos et al. forthcoming). From this material, 13 animal bones identified as *Ovis/Capra* or *Bos* were selected for SI comparison (Online Resource 2).

### Micro-remains analysis

Micro-remains were extracted from HDC to provide further information about the vegetal component of the diet, targeting phytoliths and starch granules but also documenting non-pollen palynomorphs (NPP), diatoms, and other finds of interest. Phytoliths are microfossils (2–100 μm) that form when plants deposit silica (SiO_2_·nH_2_O) in and between cells, creating a cast. This allows phytoliths to be used to identify the biological tissue in which they formed and often the plant taxa that produced them (Cabanes 2020; Mulholland et al. 1992; Piperno 2006). Phytoliths are abundant in monocotyledonous plants (hereafter monocots) and especially in the Poaceae family (grasses, including cereals), while in other angiosperm clades such as magnoliids and eudicots (including most fruits, vegetables, and woody plants) they are produced in lower numbers. Due to the redundancy of phytolith morphotypes, phytoliths provide limited information regarding specific plant species or genera, but they are useful in differentiating between plants following C_3_ versus C_4_ photosynthetic pathways (Piperno 2006). By attributing phytoliths to the plant parts in which they form, further information regarding their use can be gleaned (e.g., the use of leaves and stems for matting, inflorescence phytoliths pointing to crop or food processing, etc.) (Rosen 1992). Being very durable, phytoliths usually preserve well in archaeological contexts, including in HDC (e.g., Scott et al. 2021).

Starch granules are the basic energy storage molecules produced by many plants. In plants, starch is often stored for long-term use in storage organs such as tubers, rhizomes, bulbs, seeds, and fruits (Henry 2020). Rich in energy, starchy plant parts are sought after by humans, animals, and even bacteria, and thus are often missing from the archaeological record (Hutschenreuther et al. 2017). However, HDC and some tool surfaces have been shown to be good micro-environments for starch preservation as they provide a stable protected environment (Henry 2020). If present, starch granules can usually be identified to genera. Starch granules found in dental calculus provide information on plant consumption during an individual's life. However, starches are easily transported by air or touch, and careful measures must be taken to prevent sample contamination (Crowther et al. 2014). In addition, the low amount of starch granules that are usually recovered from each individual, and our incomplete understanding of the entrapment and preservation mechanisms in HDC, limit our interpretation (Bartholdy and Henry 2022; Leonard et al. 2015).

Diatoms are siliceous unicellular algae found in all aquatic ecosystems, including moist soils (Stone and Yost 2020), and due to a similar composition, they are often found during phytolith analysis. Non-pollen palynomorphs (NPPs) are a diverse group of microfossils (about 10–250 μm in size) found in palynological preparations other than pollen grains, mostly consisting of fungal spores, algal remains, parasite eggs, and other plant or animal micro-remains (Shumilovskikh and van Geel 2020). NPPs are usually regarded as indicators of the local environment, given their normally limited range of dispersal (Van Geel et al. 2003).

HDC sampling was carried out at the Zentrum für Anatomie of the University of Göttingen. Dental calculus was carefully removed from the surface of each tooth using dental scalers/curettes. Tools were sterilized with a diluted commercial bleach solution (1:10) and subsequently cleaned with UV-irradiated deionized water between samples to minimize cross-contamination. Calculus was scraped directly into Eppendorf tubes (1.5 ml). Nitrile gloves were worn throughout the entire process. HDC was collected per individual, meaning that calculus samples from different teeth of the same individual were combined into one tube. The limited calculus preservation on the teeth of most ancient individuals from Kamid el-Loz hindered an approach by tooth or by surface area. In addition, the calculus from Kamid el-Loz was used for a number of different analyses apart from the here presented analysis of micro-remains, so by pooling the calculus per individual, we could ensure optimal sample distribution between these different analyses and minimum sample loss.

Extraction of microremains from HDC was carried out following a lab cleaning protocol that included wiping all surfaces with 5% NaOH and collecting random contamination control samples from work surfaces (Crowther et al. 2014; Henry 2020). The HDC was treated using 500 µl of ethylenediaminetetraacetic acid solution (EDTA) pH 8.0 ~ 0.5 M in H_2_O for 48–96 h (depending on sample size). Once the dental calculus appeared mostly decalcified the samples were centrifuged at 6000 rpm for seven minutes and the supernatant was pipetted out. 200 µl of distilled H_2_O was added to the samples to wash the leftover EDTA and the samples were centrifuged again at 6000 rpm for seven minutes. Most of the supernatant was then pipetted out leaving a few µl which were mounted on several slides in a ratio of 10 µl dH_2_O and 10 µl glycerin 20%, which were then mixed and covered with an 18 × 18 mm cover slide. Slides were examined under plane and cross polarized light (PPL and XPL respectively) at 400 × magnification using an Olympus BX53 microscope. The entire slide was examined, and each microfossil was photographed, described, and documented. Phytolith and starch morphologies were identified following the standard literature as well as the starch reference collection of Leiden University (Ahituv and Henry 2022; ICPT 2019; Madella et al. 2005; Mulholland et al. 1992; Piperno 2006). Diatoms and NPPs were not the main focus of the study and therefore were also photographed at 400 × magnification, instead of at 600–1000 × magnifications usually required for more detailed identification.

### Stable isotope analysis

Stable carbon and nitrogen isotope analysis of bone collagen is a well-established method for reconstructing past diets from archaeological animal and human bone collagen (Schoeninger 2010; Richards 2020). This approach can provide information on long-term diet and help differentiate between the consumption of C_3_ and C_4_ plants, as well as the trophic level of a consumer (e.g., Sołtysiak and Fernandes 2021; Somerville and Beasley 2023). Plants with different photosynthetic pathways have different stable carbon isotope values. For example, C_3_ plants, which include the majority of trees, shrubs, and temperate grasses, as well as important domesticated crops such as wheat, barley, and rice, have more negative *δ*^13^C values (–33 to –23‰) than C_4_ plants (–16 to –9‰), which include the majority of tropical grasses and certain domesticated crops such as millet (O’Leary 1981). Marine plants generally follow the C_3_ photosynthetic pathway but typically exhibit higher *δ*^13^C values (i.e., –22 to –17‰) relative to their terrestrial counterparts due to the incorporation of ^13^C enriched ocean carbonate (DeNiro and Epstein 1978; Keegan and DeNiro 1988). Nitrogen isotopes can be used to reconstruct animal protein contributions to the diet. There is a ~ 3–5‰ difference between the *δ*^15^N values of prey and their predators, with marine consumers having generally higher *δ*^15^N values than terrestrial consumers due to longer food chains (Schoeninger and DeNiro 1984). Marine consumers also have more positive *δ*^13^C values, more similar to consumers of C_4_ resources (Ambrose and Norr 1993; Schoeninger and DeNiro 1984;). Due to fluctuating *δ*^13^C values in freshwater plants, the *δ*^13^C values from bones of freshwater fish range widely from –13 to –25‰. This variability is likely to lead to diverse *δ*^13^C values among the consumers of freshwater fish (Katzenberg and Weber 1999).

Bone samples were processed following a modified Longin (1971) method described in Richards and Hedges (1999) at the Max Planck Institute of Geoanthropology, Jena, Germany. The outer surface of the bones was first cleaned using a sandblaster with aluminum oxide sand. Roughly 500 mg of bone sample was removed using a Dremel drill and transferred to sample tubes. Samples were demineralized by immersion in 10 ml of 0.5 M HCl. The HCl solution was changed every 48 h until samples were fully demineralized. Once demineralized, samples were rinsed three times with ultra-pure water. Samples were then gelatinized in a pH 3 HCl solution at 70 °C for 48 h. The gelatinized collagen solution was then EZEE filtered. Samples were then frozen overnight and freeze-dried for 48 h or until fully dry. Roughly 1.0 mg of the sample was weighed in duplicate into tin capsules for analysis.

Stable carbon and nitrogen isotope values were measured using a Thermo Fisher Scientific Flash 2000 Elemental Analyser coupled to a Thermo Fisher Scientific Delta V Advantage mass spectrometer at the Max Planck Institute for Geoanthropology. Isotopic values are reported as the ratio of the heavier isotope to the lighter isotope (^13^C/^12^C or ^15^N/^14^N) as *δ* values in parts per mille (‰) relative to international standards, VPDB for *δ*^13^C and atmospheric N_2_ (AIR) for *δ*^15^N using the following equation [*δ* = (R_sample_ – R_standard_)/R_standard_] (McKinney et al. 1950). Results were calibrated using a two-point calibration against international standards IAEA-CH-6 (*δ*^13^C = –10.4‰), IAEA-N-2 (*δ*^15^N = 20.3‰) and USGS40 (*δ*^13^C = –4.5‰, *δ*^15^N = –26.4‰). A Sigma Aldrich fish gelatine standard (*δ*^13^C = –15.6‰, *δ*^15^N = 14.5‰) was run as an in-house standard. Replicate precision of standards was used to calculate machine error, resulting in 0.2‰ for nitrogen and 0.2‰ for carbon. Analytical error higher than 1*δ* ± 0.3‰ was not accepted.

The individuals with results passing the quality control criteria were divided into sub-groups according to period, age at death, and biological sex in order to assess differences and similarities in their mean values (Vaiglova et al. 2023). Biological sex and age at death designations were based on their assignments in the original publications (Kunter 1977; Miron 1982).

Finally, we attempted to explore the relationship between diet and burial goods, dividing the burials into categories based on the presence, quantity, and quality of grave goods. Burials with no or only 1–2 ceramic vessels were defined as “Low”, burials with animal bones, mother of pearl, beads, etc., and/or more than two ceramic artifacts were defined as “Medium”, burials with one metal object (e.g., a pair of bronze earrings) were defined as “Medium/High”, and burials with one precious metal object (silver, iron) and/or more than one bronze object together with other artifacts (beads, faience, etc.) were defined as “High” (Online Resource 1). The use of the terms “Low” vs. “High” in this case is only considered relative to each other rather than actual economic status, as we only sampled a subset of the population, and the burials are unlikely immediately contemporaneous. To avoid an age bias, we evaluated individual's diet according to burial groups only among the adults. To avoid further biases arising from grave robbery or post-depositional disturbances, we compared only the graves that were not described as disturbed by the excavators (Kunter 1977; Miron 1982). This resulted in three burials from three different categories from the MBA II, and seven “Low”, four “Medium” and 21 “High” burials from the IA III / Persian-Hellenistic cemetery (Table 1).

## Results

### Micro-remains analysis

Dietary plant micro-remains (phytoliths and starch granules) were found in all HDC samples in varying numbers regardless of their archaeological period (Online Resource 3, Fig. 3). Starch preservation was low with a total of ten granules found in the HDC of six different individuals: one MBA II individual (G_97), and five from the IA III / Persian-Hellenistic periods (G_15, G_48, G_66, G_71, and G_IIE 7:1). Five starch granules were extremely eroded, and another was too small to be identified (Fig. 4a-b). The other four starch granules were assigned to three different types.

**Fig. 3:**
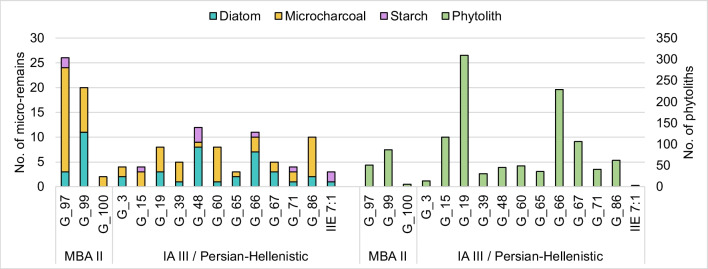
A chart showing the numbers of the different micro-remains in each dental calculus sample. The phytoliths (in green) correspond to the secondary axis on the right

**Fig. 4:**
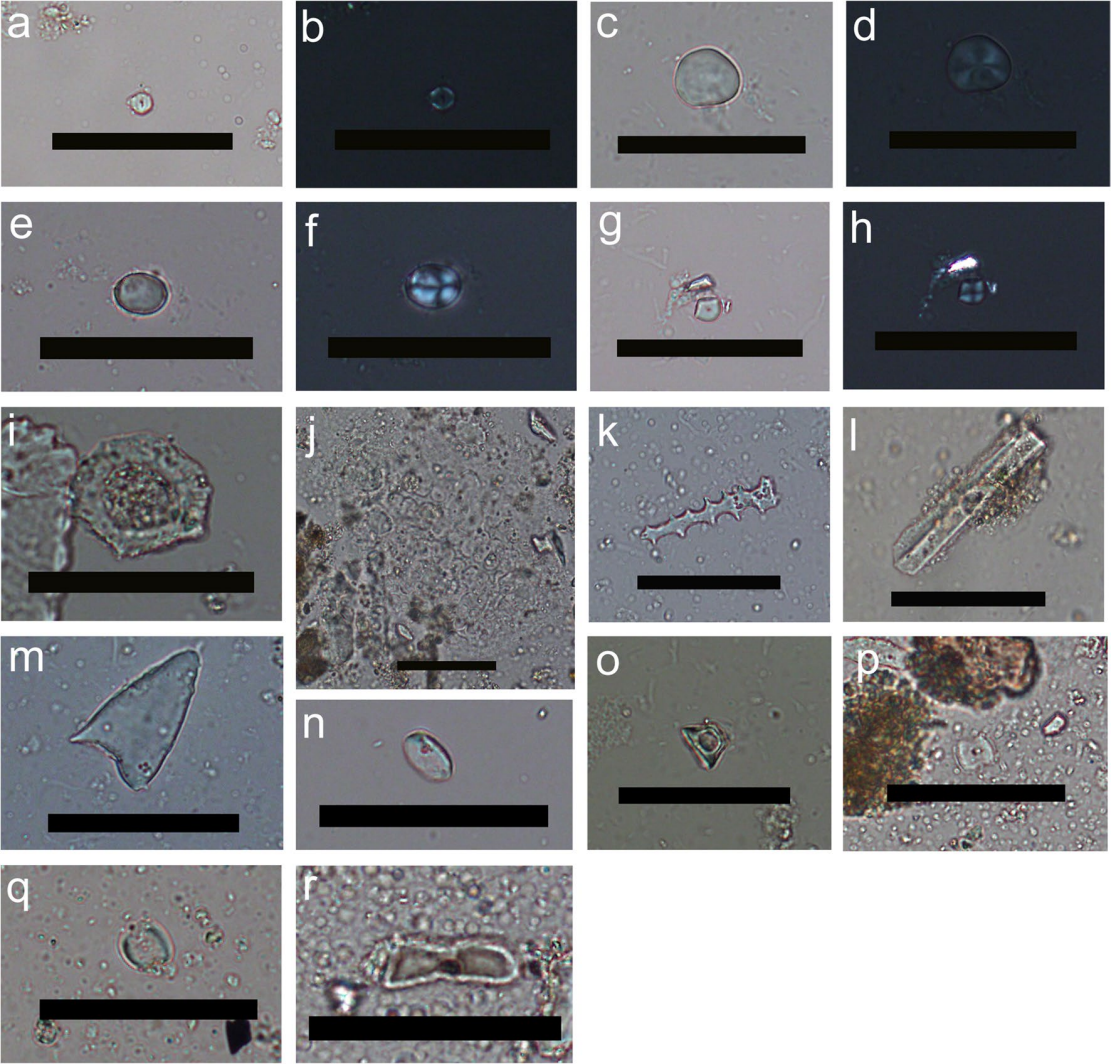
Plant micro-remains from Kamid el-Loz HDC. **a**-**b**. Unidentified small starch, typical of many grass species, under PPL and XPL; **c**-**d**. Type 1 starch identified as a TASH starch under PPL and XPL; **e**–**f**. Type 2 starch possibly originating from a USO, under PPL and XPL; **g**-**h**. Type 3 starch, possibly derived from an AP grass, under PPL and XPL; **i**. Cyperaceae achene phytolith under PPL; **j**. Grass inflorescence multi-cell with elongate dendritic and papillae under PPL; **k**. Long cell echinate under PPL; **l**. Elongate parallelepiped under PPL; **m**. Prickle under PPL; **n**. Short cell rondel “pinched top” under PPL; **o**. Short cell rondel under PPL; **p**. Short cell saddle under PPL; **q**. Short cell “squashed” saddle under PPL; Short cell bilobate under PPL. All images were taken at 400 × magnification, scale bars = 50 µm

Type 1 is a simple lenticular starch granule (17–48 × 15 µm), which is circular to subcircular in plane view and compressed with one flatter side and a rounded dome on the other side. It has very fine and faint central lamellas, indistinct hilum, and diffused almost centric cross in XPL (Fig. 4c-d). Type 1 is confidently identified as TASH (*Triticum*, *Aegilops*, *Secale*, or *Hordeum*). Two Type 1 granules were found: one damaged granule tentatively identified as Type 1 starch in the HDC of an MBA II individual (G_97), and a well-preserved granule in the HDC of IA III / Persian-Hellenistic individual (IIE 7:1).

Type 2 is a small (12 × 9 µm) simple oval granule with an eccentric hilum and a distinct cross under XPL with thin arms that are either symmetric or asymmetric depending on the view (Fig. 4e-f). This starch granule is very tentatively assigned to an unidentified Underground Storage Organ (USO) such as bulbs, tubers, and rhizomes (e.g., Type 7 in Santiago-Marrero et al. 2021). One Type 2 granule was found in the HDC of IA III / Persian-Hellenistic individual G_15.

Type 3 is a simple small (⌀8 µm) hemispherical granule with two facets. It has a central refractive hilum with a centric cross with thin arms under XPL (Fig. 4g-h). Although this type can be assigned to many grasses, the faceted appearance suggests that it originated from an AP grass (Paniceae [e.g., millet] or Andropogoneae [e.g., sorghum]). One Type 3 granule was found in the HDC of IA III / Persian-Hellenistic individual IIE 7:1.

**Fig. 5:**
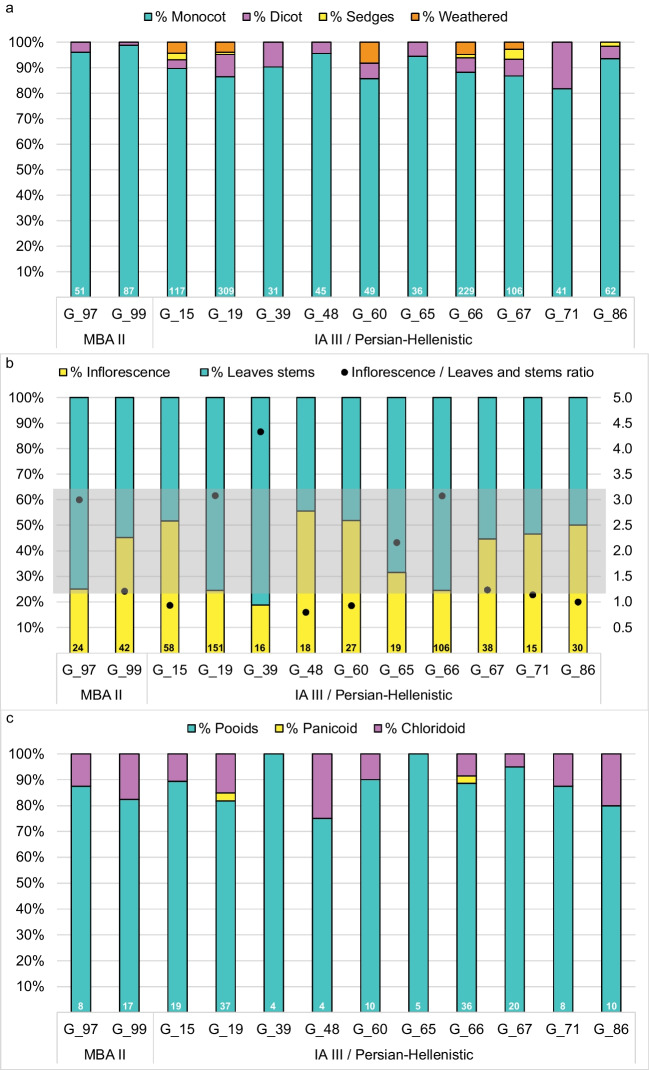
**a**. A chart showing the percentages of phytoliths attributed to monocots, dicots, sedges, and weathered morphotypes out of the total number of identifiable phytoliths in each sample; **b**. Percentages of inflorescence vs. leaf and steam from the total monocots phytoliths that can be assigned to plant parts. On the secondary axis, the leaf/stem to inflorescence ratio is presented as black circles. The grey area represents the range of the ratio in whole plants of several wild and domesticated plants presented in Regev et al. (2015). Values below this area represents assemblages dominated by inflorescence phytoliths; c. Percentages of the grass short cell phytoliths of the three Poaceae subfamilies from the total number of grass short cells in each sample. The total number of phytoliths per sample used for each calculation is provided at the bottom of each bar

Phytoliths were found in all samples, totaling 1185 phytoliths (Online Resource 3, Fig. 3). Their abundance in specific samples ranged from 6 to 307. The higher amounts exceed numbers typically found in prehistoric dental calculus studies from the ANE (Hart 2014; Hardy et al. 2016; Henry et al. 2011), but they are in line, or even lower than, the number of phytoliths identified by Scott et al. (2021) for HDC samples from LBA Megiddo and Iron Age Tel Erani from the Southern Levant. Three of the samples (G_100 dated to the MBA II and G_3 and G_IIE 7:1 dated to the IA III / Persian-Hellenistic) contain too few phytoliths to allow further interpretation and are excluded from the following description. The other 12 phytolith assemblages are characterized by high percentages of phytoliths derived from monocots (between 82 and 99%) and low percentages of dicot phytoliths (1–18%), Cyperaceae-specific phytoliths (sedges 0–4%), and weathered phytoliths (0–8.2%) (Fig. 4i, 5a). High percentages of monocot phytoliths are common in many archaeological samples (e.g., Scott et al. 2021) as monocots, and especially the Poaceae family (grasses, including cereals), are prolific phytolith producers. As Arecaceae (palm) phytoliths were not found in any of the samples, the monocot phytoliths likely originated from grasses and sedges (Albert et al. 2008). In contrast to monocots, dicots and other angiosperms (including most fruits and vegetables) tend to produce few phytoliths and therefore are underrepresented. It should be noted that six of the HDC assemblages from the IA III / Persian-Hellenistic individuals also contain Cyperaceae phytoliths (Figs. 4i, 5a, Table 2, note that sample G_86 does not appear in the chart), including sedge achene phytoliths (0–4%). The assemblages from the later periods also contained weathered phytoliths (0–8%), while the samples from the MBA II have neither.

There is no clear pattern in the percentages of monocot phytoliths originating from inflorescence vs. leaves and stems (Fig. 4j-m, 5b). We examined the ratio of the leaf and stem to inflorescence phytoliths (0.8–3.1) and compared it to the ratios published by Regev et al. (2015) for whole plants samples of various domesticated and wild species, which usually produce more leaves and stems phytoliths (1.2–3.2) (Fig. 5b). While half of the assemblages from Kamid el-Loz individuals fall within the range of a whole plant (n = 6), only one of the remaining half is dominated by leaf and stem phytoliths. The other samples (n = 5) with a ratio lower than 1.2, show a tendency toward having more inflorescence than leaf/stem phytoliths. A similar pattern was also identified in the HDC at two other sites in the Southern Levant (MBA III—LBA I burials at Megiddo and Iron Age Tel Erani, see Scott et al. 2021).

The grass short cell phytoliths in all samples are dominated by C_3_ Pooideae subfamily grass forms (rondels, trapeziform, and creanate morphotypes, 75–100%, Fig. 4n-o and 5c). Pooideae grasses of this subfamily (including wheat, barley and oat) grow in cool and humid conditions. All samples except two contain low percentages of saddle short cells (Fig. 4p-q) which are usually attributed to the C_4_ Chloridoideae subfamily (0–25%) which thrive in warm and dry climatic conditions (i.e., *Eleusine indica* (goosegrass); *Eleusine coracana (*finger millet)). Finally, two of the individuals (G_66 and G_19) from the IA III / Persian-Hellenistic period have low percentages (3.0%) of bilobate and polylobate short cells (Fig. 4r) which are usually attributed to the C_4_ Panicodeae subfamily and thrive in warm and humid environments. It is important to note that due to the presence of Cyperaceae phytoliths, it is possible that the saddles and bilobate/polylobates are derived from *Arundo donax* and *Phragmites communis* respectively, which grow in wetlands or riparian habitats. The perennial spring directly to the north of the tell or the seasonal spring at the tell’s western edge would have provided suitable habitats for these plants (Hachmann 1989, 14). Furthermore, well into modern times, large parts of the southern Beqa’a plain turned into seasonal marshes during the winter months. These seasonal wetlands are also historically attested, and their southernmost tip is located ca. 5 km to the north of Tell Kamid el-Loz. Hachmann also presents archaeological and historical evidence that suggests the existence of a lake in these areas in ancient times (Hachmann 1989, 20–23). Similar ratios of grass short cells were recorded in samples from Megiddo and Tel Erani further south (Scott et al. 2021).

**Fig. 6:**
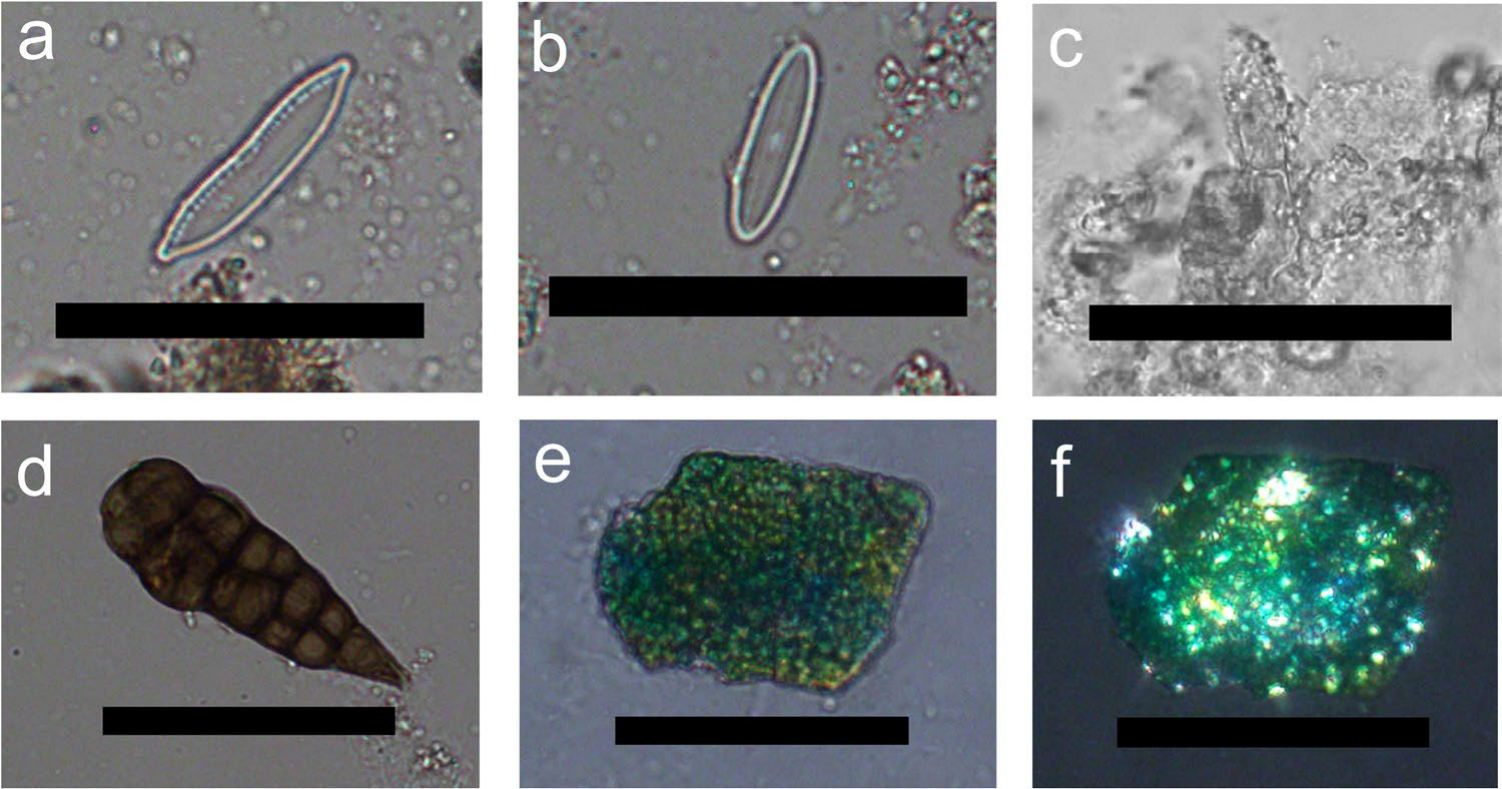
Different micro-remains found in HDC samples: **a**. *Hantzschia* sp. diatom (G_99); **b**. Possible genus *Navicula* diatom (G_99); **c**. possible genus *Aulacoseira* diatom (G_39); **d**. *Alternaria* sp. fungal genus (G_48); **e**–**f**. Green–blue mineral grain under PPL and XPL (G_71). All scales are 50 µm

The diatoms present were mostly identified as freshwater diatoms, although identification was not always possible due to the low resolution of 400 × magnification (Fig. 6a-c). Almost all the diatoms identified were *Hantzschia* sp. (n = 26/36), a genus known to live in soils. Other genera tentatively identified include *Navicula* sp. (n = 2/36), which are motile benthic diatoms living attached to substrates in shallow water, and *Aulacoseira* sp. (n = 5/36), plankton species found in inland water bodies. Diatoms tentatively identified as belonging to *Navicula* and *Aulacoseira* were all highly dissolved and not definitive. The remaining diatoms were represented by unidentifiable fragments.

Non-pollen palynomorphs (NPPs) were mostly identified as amorphous fragments of plant material. One sample from the IA III / Persian-Hellenistic cemetery (G_48) contained spores of *Alternaria* sp., a parasitic fungal genus that feeds on fruits and vegetables, often attacking cereals (Ellis and Ellis 1985; Gibaja et al. 2023; Mazzucco et al. 2022) (Fig. 6d). The lab control sample contained a *Capillaria* sp. egg from an intestinal parasite (Morandi 2018) likely originating from the animal dung regularly analyzed in the lab. The control sample did not contain any starch granules or phytoliths, indicating that the finds in the archaeological samples did not originate from contamination during laboratory processing. A final finding of interest includes green–blue angular inclusions that we were not able to securely identify. These appeared in the HDC of several IA III / Persian-Hellenistic individuals (Fig. 6e-f) but were absent from control samples collected from the laboratory working surfaces. An attempt to identify their composition using Fourier Transform Infra-Red microscopy (µFTIR) was not successful. Considering their grainy appearance, angular shape, and interference colors, they resemble a micritic mineral. Integration of different non-dietary particles into HDC is not uncommon and can come from many sources (Radini et al. 2017).

### Stable isotope analysis

A total of 94 human rib bones (12 from MBA II individuals and 82 from IA III / Persian-Hellenistic individuals) and 13 LBA animal bones (7 *Ovis/Capra*, and 6 *Bos*) had enough collagen for stable isotope analysis (Online Resource 2). Based on previous studies (DeNiro 1985; Guiry and Szpak 2021; Vaiglova et al. 2023; Van Klinken 1999), several quality control criteria such as collagen wt%, C wt%, N wt%, and C:N ratio were used to assess the collagen preservation and contamination and to select better-preserved bones for diet reconstruction. The wt% collagen yields were variable (between 1.3–18.4%, with a mean of 7.0 ± 3.8%), but none of the bones had less than 1% collagen yields which, according to Van Klinken (1999), would point to collagen degradation. C:N ratios were between 3.1 and 3.5, falling within the range for fresh mammal bones (3.3 ± 0.3, Van Klinken 1999) and the range of 2.9 and 3.6 shown by DeNiro (1985) to be acceptable for archaeological bones. Van Klinken reported wt% C in the range of 35 ± 8.8%, with higher percentages likely pointing to the presence of organic carbon contaminants, and 11%–16% for wt% N.

Following Ambrose (1990) and Ambrose and Norr (1992), we used 15–47% as the cutoff range for wt% C and 5–17% for wt% N, similar to Stantis et al. (2022), who analyzed bones from the MB layers at Sidon on the Lebanese coast. The wt% C of the samples from Kamid el-Loz ranged from 22.5–51.1%, averaging 43.5 ± 5.0%. The wt% N of the samples ranged from 8.2–17.6%, averaging 15.3 ± 1.7%. Any measurements above 47% wt% C or 17% wt% N were discarded. Finally, any set of duplicate measurements where the standard deviation of the %C and %N was > 2.0% were discarded for possible contamination or preservation issues, leaving us with a total of 87 bones: 10 from the MBA II, 64 from the IA III / Persian-Hellenistic Periods cemetery, 7 LBA *Ovis/Capra*, and 6 LBA *Bos* (Online Resource 4). Archaeological data on the burials (Miron 1982; Poppa 1978) was used to further divide the individuals according to sex, age, and burial goods (see Sect. "Stable isotope analysis").

**Fig. 7:**
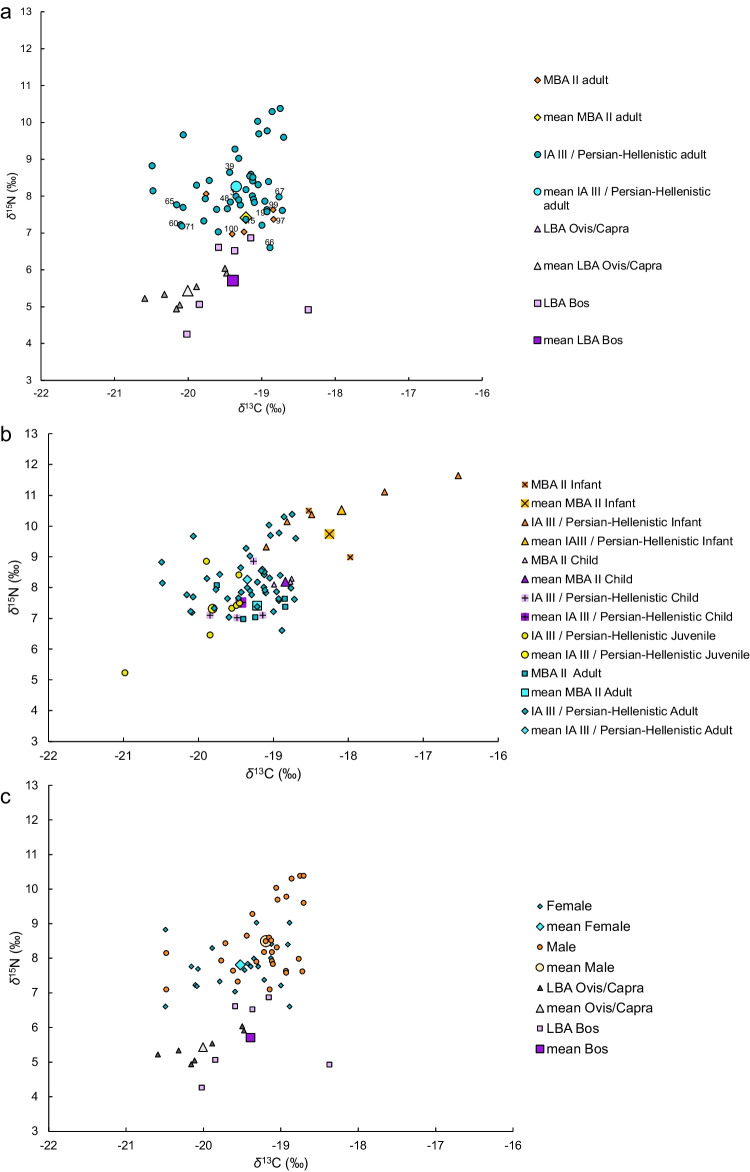
**a**. Scatter plot of *δ*^13^C and *δ*^15^N values of adult humans from the MBA II and IA III / Persian-Hellenistic periods and animal bones from the LBA. The mean of each group is presented. Individuals who were also analyzed for HDC are labeled with grave numbers. **b**. Scatter plot of *δ*^13^C and *δ*^15^N value of human from the MBA II and IA III / Persian-Hellenistic divided according to age groups. The mean of each group is presented. **c**. Scatter plot of *δ*^13^C and *δ*^15^N value of human from the IA III / Persian-Hellenistic divided according to estimated sex, and animal bones from the LBA. The mean of each group is also presented

To compare the *δ*^13^C and *δ*^15^N results of the different groups (below) we first studied their distribution using the Shapiro–Wilk test and histograms to check for normality. As most of the groups were not normally distributed, we used either the Kruskal–Wallis test to compare more than two groups (e.g., when comparing age groups or burial goods) or the Wilcoxon Two-Sample test when comparing two groups (e.g., by period or sex) (Table 3). The scatterplot of Kamid el-Loz MBA II and IA III / Persian-Hellenistic adult humans and LBA ovicaprines and cattle bone collagen *δ*^13^C and *δ*^15^N values (Fig. 7a) show values aligning with reliance on terrestrial C_3_ plants protein input for humans and animals and for the humans also protein intake from terrestrial animals who fed on C_3_ plants. The *Ovis/Capra* bones have *δ*^13^C values between –20.6 and –19.5‰ with a mean of –20.0 ± 0.4‰ and *δ*^15^N values in the range of 4.9–6.0‰ with a mean of 5.4 ± 0.4‰. The *Bos* samples have *δ*^13^C values between –20.0 and –18.4‰ with a mean of –19.4 ± 0.6‰ and *δ*^15^N values between 4.3–6.9‰ with a mean of 5.7 ± 1.1‰.

The adult human bones have a *δ*^13^C range of –19.8 to –18.8‰ with a mean of –19.2 ± 0.4‰ for the MBA II and between –20.5 and –18.7‰ with a mean of –19.4 ± 0.5‰ for the later individuals. The *δ*^15^N values of the MBA II individuals are between 7.0 to 8.1‰ with a mean of 7.4 ± 0.5.0‰ and a range between 6.6 to 10.4‰ with a mean of 8.3 ± 0.9‰ for the IA III / Persian-Hellenistic periods. The adult human bones from the MBA II and from the IA III / Persian-Hellenistic periods, show statistically significant differences in *δ*^15^N values, but no significant differences in *δ*^13^C values (Table 3).

Looking at the *δ*^15^N results according to age groups (Fig. 7b), we see that two of the age groups have individuals on opposite sides of the dietary range, with several of the individuals from the infant group having more positive *δ*^13^C and *δ*^15^N values relative to the rest of the population (mean of 9.7 ± 1.1‰ for the MBA II and 10.5 ± 0.9‰ for the IA III / Persian-Hellenistic, see also Table 3), and two of the individuals in the juvenile group having more negative *δ*^13^C and lower *δ*^15^N values comparing to the rest of the population (mean of –19.8 ± 0.6‰ for *δ*^13^C and 7.3 ± 1.2‰ for *δ*^15^N, Table 3, Fig. 7b). While the age groups from the MBA II period were too small for statistical analysis, the infant group from the IA III / Persian-Hellenistic is significantly different from the rest of the population both in *δ*^13^C and *δ*^15^N values (Table 3).

The stable isotope values of adult females and adult males from the IA III / Persian-Hellenistic periods were also compared (Fig. 7c, Table 3). The statistical analysis shows significant differences in *δ*^13^C and *δ*^15^N values between the two groups. Looking at the scatter plot (Fig. 7c), it is evident that although there are similar values, the males have more positive *δ*^13^C values (with a mean of –19.2 ± 0.4‰ compared to a mean of –19.5 ± 0.5‰ for the female) and higher mean values of *δ*^15^N (8.5 ± 1.0‰ compared to 7.8 ± 0.6‰ for the female). The range of *δ*^15^N values of the male group is also larger than that of the female (3.3 and 2.4‰ respectively).

The *δ*^13^C and *δ*^15^N values of MBA II and IA III / Persian-Hellenistic adults according to burial goods are presented in Table 3, and Online Resource 5b. Note that statistical analysis was performed only on the IA III / Persian-Hellenistic Periods adults as the MBA II sample size was too small. The statistical analysis showed no correlation between grave goods and SI values. The scatter plot also shows no significant differences in *δ*^13^C and *δ*^15^N values between the groups.

## Discussion

### Reconstructing dietary practices and the use of plants dietary resources at Tell Kamid el-Loz over time

The preservation of plant micro-remains in the Kamid el-Loz HDC samples is moderate. There is a generally high number of phytoliths in most samples, low percentages of weathered phytoliths (0.0–8.6%), and high percentages of long cells (64.3–79.7% Online Resource 3). However, many of the long cells were broken, and the number of phytoliths in anatomical connection was rather low (for phytolith preservation criteria see Madella and Lancelotti 2012). Starch granules are uncommon, yet of the ten granules identified, four are well-preserved, and one is identifiable despite being damaged. This may suggest that the plant micro-remains were not heavily affected by chemical degradation, but rather by both mechanical degradation and depositional processes. It is still unclear what mechanisms lead to the entrapment, preservation, and degradation of plant micro-remains in dental calculus (Bartholdy and Henry 2022). We know, for example, from experimental and ethnoarchaeological studies, that the micro-remains found in HDC are a mere fraction of the consumed diet, and that there is a size bias in the incorporation of micro-remains into the biofilm matrix (Bartholdy and Henry 2022; Leonard et al. 2015; Power et al. 2015, 2021). Leonard et al. (2015), who compared the plant micro-remains in HDC of a forager-horticulturalists population in Tanzania with their recorded diet, showed that there is a large individual variation in plant representation that does not necessarily indicate variation in plant consumption, and that at least 50 individuals are needed in order to capture the full range of diet within a population. Therefore, due to the small sample size, we must be cautious when trying to broadly interpret the HDC micro-remain record from Kamid el-Loz. Regardless, we assume that HDC microremains originate from plants which were introduced into the mouth, most likely as part of the diet.

The starch and phytolith micro-remains nevertheless indicate that, during the MBA II Period, the inhabitants of Tell Kamid el-Loz consumed cereals. Starting from the IA III / Persian-Hellenistic periods, we also see evidence for the consumption of small-grained panicoid grasses, and underground storage organs. We cannot rule out the possibility that these types of plants were consumed earlier. The grass short-cell phytoliths indicate that the majority of plants introduced to the mouth across all time periods were C_3_ plants, as evidenced by the high percentages of rondel and trapeziform morphotypes of the Pooideae subfamily. Additionally, some contribution of C_4_ plants from the Chloridoideae subfamily are evidenced by saddle short cells (Twiss et al. 1969). This agrees with the SI results (Sect. "Reconstructing changes in dietary practices at Tell Kamid el-Loz over time using SI analysis"). In the HDC of the IA III / Persian-Hellenistic individuals, we have some evidence for plants from the Panicoideae subfamily, indicated by the presence of bilobate short cells and a possible AP starch granule. As no long cell phytoliths typical to Panicoideae cereals such as millet were identified (cf. Laugier et al. 2022), it is hard to attribute it to specific plant taxa with certainty. Cyperaceae phytoliths, and specifically phytoliths originating from Cyperaceae nutlets, are present. Many species of Cyperaceae are edible, and evidence for the consumption of Cyperaceae rhizomes and nutlets is found across the Mediterranean from different time periods, including at the Phoenician settlement at Tell el-Burak (Crawford 2007; Fox et al. 1996; Orendi and Deckers 2018; Wollstonecroft et al. 2011). The presence of Cyperaceae phytoliths indicates that the inhabitants of Kamid el-Loz were utilizing plants from aquatic environments, raising the possibility that some of the saddle short cells could have originated from reeds (Arundinoideae subfamily) rather than the Chloridoideae subfamily. Still, even in the IA III / Persian-Hellenistic periods, C_4_ plants made up only a small contribution to the diet, with both the HDC and SI data pointing to an overall reliance on C_3_ plants.

Phytolith assemblages found in HDC from two sites in the Southern Levant (MBA III—LBA I burials at Megiddo and Iron Age Tel Erani, see Scott et al. 2021) also have a high abundance of leaf and stem phytoliths compared to inflorescence phytoliths, contrary to studies of prehistoric populations (e.g., Hardy et al. 2016; Hart 2014; Henry and Piperno 2008; Cummings et al. 2018). One might ask why are there so many phytoliths in human dental calculus, as phytoliths are more abundant in the less edible parts of the plants such as the leaves, stems, and husks, while they are absent in the more edible parts such as grains, vegetables, and fruits. A general pattern across the region at this period suggests that there may be a broader cultural pattern in which phytolith-rich plant parts were regularly introduced to the mouth, either as part of the diet or for other reasons. Several possible explanations can be considered: 1) that the phytoliths originated from the consumption of cereal-based foods or drinks that were not well refined (e.g., bread made of flour that was not sieved well, unfiltered beer); 2) the phytoliths originated from the consumption of other plant parts of grasses, such as shoots; 3) the use of plant-made utensils (e.g. drinking straws, “toothbrushes”, etc.) that introduced the phytoliths into the mouth; 4) using the mouth as an additional “hand” for work-related activities such as basket weaving, sewing, etc. The presence of the blue-green mineral grains also hints at non-dietary pathways for the accumulation of other particles in the dental calculus, such as the introduction of mineral debris from food processing with grinding stones, the use of glazed vessels, or the processing of pigments. Despite the possibility for the introduction of additional phytolith that are not directly related to diet, the presence of the Cyperaceae nutlets phytoliths and starch granules, indicate that plant consumption is the most likely scenario for micro-remains entrapped in the HDC. In addition, as this increase in phytolith numbers in HDC from the region is so far attested only in agrarian societies, we would like to suggest the hypothesis that it is related mostly to the increase in cereal consumption following their domestication. Additional studies of plant micro-remains in HDC from the region are called for to test this hypothesis.

### Reconstructing changes in dietary practices at Tell Kamid el-Loz over time using SI analysis

The SI results indicate that the diet of the inhabitants of Kamid el-Loz relied on C_3_ crops and domesticated animals that were also feeding on C_3_ plants, a diet typical for Levantine farmers (Stantis et al. 2022; Zohary et al. 2012). Comparable results are available from the MBA site of Sidon on the Mediterranean coast (Schutkowski and Ogden 2011; Stantis et al. 2022). Based on archaeozoological data, such a diet included the consumption of ovicaprines, cattle, and pigs, as well as some wild game animals (Bökönyi 1990). As evident from macro-botanical remains, wheat, barley, chickpea, bitter vetch, sweet pea, lentils, and wine grapes were some of the most common food crops in the area (Behre 1970). Beginning in the Iron Age, millet (*Panicum miliaceum* and *Setaria italica*) consumption became more common (Riehl and Nesbitt 2003, but see Laugier et al. 2022 for the problematic nature of millet identification from macro-botanical remains).

**Fig. 8:**
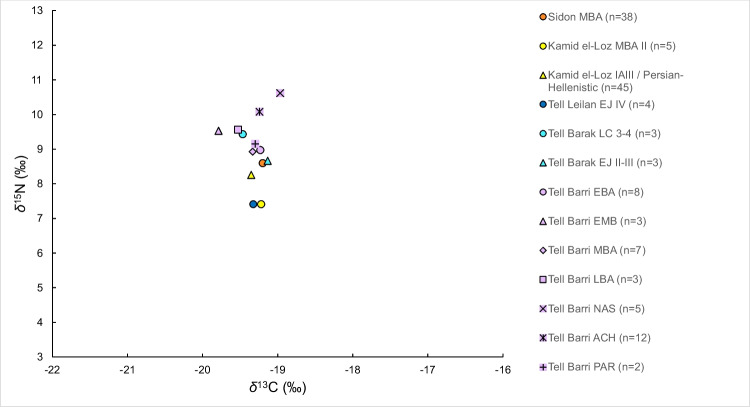
Scatter plot of the mean *δ*^13^C and *δ*^15^N values of adults from North Syria Tell Brak (LC = Late Chalcolithic, c. 3900–3300 cal BCE, EJ II-III = Early Jezireh II-III = EBA, c. 3000–2000 cal BCE), Tell Barri (EBA = c. 2800–2200 BCE, EMB = Early/Middle Bronze Age, c. 2200–2000 BCE, MBA = c. 2000–1500 BCE, LBA = c.1500–1200 BCE, NAS = Neo-Assyrian Period, c. 900–800 BCE, ACH = Achaemenid Period c. 500–300 BCE, PAR = Parthian Period, c.100–300 CE), and Tell Leilan (EJ IV = EBA IV, c. 2300–2230 BCE). Two sites from the Northern Levant; Sidon (MBA, c. 2000–1600 BCE) and Kamid el-Loz (MBA II c. 1750–1550 BCE, IAIII / Persian Hellenistic c. 538–60/30 BCE)

We compared the SI results from Tell Kamid el-Loz to the few existing previous studies of diet using SI analysis of adults bone collagen from Near Eastern sites such as from Sidon in coastal Lebanon (MBA, Schutkowski and Ogden 2011) and three sites in North Syria; Tell Brak (Late Chalcolithic and EBA II-III, Styring et al. 2017), Tell Barri (EBA- Parthian Empire period, Sołtysiak and Schutkowski 2015), Tell Leilan (EBA, Styring et al. 2017) (Fig. 8, Online Resource 6). The *δ*^13^C values of the inhabitants of all these sites are similar and point to protein input mostly from C_3_ resources. At Tell Barri a tendency to more positive *δ*^13^C values over time was identifyied (Sołtysiak and Schutkowski 2015). There is more variation in the values of *δ*^15^N between the individuals from the different sites. The individuals from EBA Tell Leilan (7.4‰) and MBA II Kamid el-Loz (7.4‰) show lower values than the individuals from IA III / Persian Hellenistic Kamid el-Loz (8.3‰), MBA Sidon (8.6‰), EBA and LC Tell Barak (8.7–9.4‰), and EBA-PAR Tell Barri (8.9–10.6‰). It is hard to point to a regional or temporal pattern that can explain this variability which can also be the result of environmental differences.

Looking at the inhabitants of Kamid el-Loz, the *δ*^15^N values for the IA III / Persian-Hellenistic adults have a wider range than those of the MBA II (Fig. 7a, Table 3). This perhaps suggests that a larger variety of protein sources contributed to the diet in the later periods, although this interpretation is limited due to the small sample size from the MBA II. Considering that some individuals have higher *δ*^15^N values that cannot be fully explained from the values of the terrestrial fauna, it is possible that some protein intake derived from aquatic sources. Freshwater fish is a more likely candidate than marine foods as there is no shift to more positive *δ*^13^C values as would be expected. The analyzed faunal remains from these periods have yielded only a small number of marine fish bones, although this may reflect excavation techniques and the lack of flotation. However, without a larger sample size from all periods and a better background of SI results of animal bone collagen from the site it is hard to suggest a secure interpretation. Despite these small differences in the range of dietary protein sources, it appears that the diet (at least as resolved through SI analysis) of the site’s inhabitants remained generally similar over time.

The SI analysis reveals some variations in the diets of different groups within the two periods. The statistically significant more positive values of *δ*^13^C and *δ*^15^N of the infant group (aged 0.6–2.5 yrs) when compared to the other age groups from the IA III / Persian-Hellenistic, most probably indicates a nursing signal as bone collagen from exclusively breastfed infants has a mean enrichment of 1‰ for *δ*^13^C and 2–3‰ for *δ*^15^N and tissues formed before the end of weaning continue to be enriched between 0.5% and 2‰ compared to maternal tissues (Chinique de Armas et al. 2022; Fuller et al. 2006). The scatter plot (Fig. 7b) suggests that individuals from the infant group during the MBA II Period may have had dietary differences compared to the rest of the population. Further isotopic studies assessing weaning practices are necessary to elucidate if there are any dietary differences between weaning infants and the larger population. The dietary practices of the juvenile and adult groups from the IA III / Persian-Hellenistic periods show statistically significant differences in *δ*^13^C (Table 3, Fig. 7b). However, this is influenced by the values of an outlier individual (Grave 24, KL68:441) with an especially C_3_-dominated plant-based diet and lower input of animal protein.

There are statistically significant differences both in *δ*^13^C and *δ*^15^N values between males and females in the IA III / Persian-Hellenistic periods, with lower values in females for both (Table 3, Fig. 7c). This may indicate sex-specific differences in diet in the IA III / Persian-Hellenistic, with males typically having more input of dietary protein from animals or possibly aquatic proteins from freshwater fish, resulting in an increase of more than 2–3‰ *δ*^15^N values than the highest *Bos* but only slightly more positive *δ*^13^C values for the same individuals. Due to a sample size of five, it was impossible to check for differences between the sexes from the MBA II population, but in the study of bone collagen at MBA Sidon, differences between the sexes were not detected (Schutkowski and Ogden 2011). At other sites in the ANE, it was impossible to statistically compare the sexes due to preservation issues or sample size. It is possible, during the later periods, that the rural population of the Beqa’a plain had some clear dietary separation between the sexes, but it is hard to say if this was a common practice or whether it reflects a change in dietary and social practices over time. Finally, we could not see statistically significant differences between adults with different burial goods, which is also in line with the findings from Sidon (Schutkowski and Ogden 2011; Stantis et al. 2022).

### Reconstructing the diet of individuals with both plant micro-remain and SI results

Of the 12 individuals who had enough plant micro-remains in their HDC for analysis, 11 yielded usable SI values (Table 2, Fig. 7a). As the HDC analysis provides only partial information on plant consumption (see above), any interpretation should be cautious. While all the Kamid el-Loz individuals have *δ*^13^C values that point to a diet based on the consumption of C_3_ plants, five individuals have more positive *δ*^13^C values compared to the means of the two periods: two from the MBA II (G_97 and G_99) and three from the IA III / Persian-Hellenistic (G_19, G_66, G_67). The two MBA II individuals have moderate amounts of Chloridoid C_4_ phytoliths in their HDC assemblages (12.5 and 17.6%). The HDC of the three individuals from the later periods had phytoliths derived from C_4_ plants such as Panicoids (G_19, G_66), and Cyperaceae which can be either C_3_ or C_4_ (G_19, G_66, G_67). Yet, the percentages of C_4_ short cells in their assemblages are high only in one sample (G_19 = 18.2%), and medium–low in the other two (11.4 and 5%). Three IA III / Persian-Hellenistic individuals with more negative *δ*^13^C relative to the mean (G_60, G_65, G_71) have medium to very low percentages of short cells derived from C_4_ plants (10, 0, and 12.5% respectively). The three IA III / Persian-Hellenistic individuals who have *δ*^13^C values similar to the mean (G_15, G_39. G_48), have very low to high percentages of C_4_ short cells (10.5, 0, and 25% respectively). Individual G_15 also has about 2.6% of phytoliths derived from Cyperaceae.

These results indicate that while phytolith short cell percentages are reliable in predicting the general contribution of C_3_/C_4_ plants to the human diet, they are not sensitive enough to be used to discern nuanced differences between individual diets at the same way SI does. It is possible that because the main contributors to the C_4_ signal are short cells from the Chloridoid subfamily which are more likely to originate from wild plants and weeds in this case (due to the lack of millet long cells), it is less reflected in the *δ*^13^C signal. Due to their low numbers and poor preservation, the starch granules can only give anecdotal information. Still, if we accept that the T2 starch in the HDC of individual G_15 originated from an underground storage organ, it adds a previously unattested plant food type to the diet of Kamid el-Loz’s ancient inhabitants. More experimental and ethnoarchaeological studies combining SI and HDC analysis will hopefully improve our ability to interpret these results in tandem.

Finally, we see that each of these methods contributes a different aspect to the reconstruction of dietary practices, and the two are not easily combined. While SI analysis provides information on broad patterns in plant and animal protein intake over the last 5–10 years in an individual’s life, it is so far unclear what period of time is represented by the plant micro-remain assemblages found in HDC. As the number of HDC samples from Kamid el-Loz is too few to reconstruct the entire range of diet of the population, it is hard to draw broad conclusions from them. Yet, due to their higher resolution, the HDC analysis does provide us with critical information about the type of environments utilized, which plant parts were introduced into the mouth, and certain plant taxa that would otherwise be invisible. Therefore, both of these methods contribute complementary information about the diet of the inhabitants of Kamid el-Loz.

## Conclusions

In this study we examined dietary changes at Tell Kamid el-Loz, between the MBA II and the IA III / Persian-Hellenistic periods, using plant micro-remains from HDC and SI analysis of bone collagen. Despite issues with a small sample size (HDC analysis), the lack of a comprehensive faunal baseline, and some preservation issues (SI analysis), we demonstrated that the inhabitants of Kamid el-Loz during the MBA II and the IA III / Persian-Hellenistic periods had similar diets. Both the SI analysis and the plant micro-remains indicate that the site’s inhabitants and animals relied mostly on C_3_ plants, and humans consumed terrestrial animal protein, possibly with increasing frequency or more varied sources in the later periods. The plant micro-remains suggest that sedges and C_4_ plants were consumed in the later periods, thus providing us with a higher taxonomic resolution and information about different environmental habitats that were utilized around the site. Our hypothesis, that changes in settlement (urban to rural) and the diachronic gap between the periods might result in dietary changes, was not particularly confirmed, although we note some differences between the two periods. Hopefully, future studies will expand on our analysis of more individuals from Kamid el-Loz and will help improve our understanding of diachronic dietary patterns.

Due to our limited understanding of how micro-remains are incorporated into HDC, and our small sample size, it is hard to draw conclusions on the contribution of plant consumption to human diet at the population level in this case. Future work targeting the incorporation pathways will help clarify how HDC micro-remains relate to plant consumption. In addition to general dietary patterns, the SI analysis provides indication for dietary differences between the sexes in the later period and demonstrated a clear nursing effect in the infant age group. Altogether, these two methods add to our understanding of the site’s inhabitants’ diet and social practices in a way that could not be achieved by utilizing only one method. Future archaeological studies on dietary practices in the region would also benefit from a similar multi-proxy approach.

### Supplementary Information

Below is the link to the electronic supplementary material.

Supplementary file1 (XLSX 18.7 KB)

Supplementary file2 (XLSX 53.3 KB)

Supplementary file3 (XLSX 24.9 KB)

Supplementary file4 (XLSX 29.6 KB)

Supplementary file5 (PDF 135 KB)

Supplementary file6 (XLSX 13.1 KB)

## Data Availability

Most of the data produced for this research is presented in this manuscript and supplementary material. Data on the animal bones from Kamid el-Loz analyzed for this research can be found in the OssoBook database which is part of the XBook Database Framework https://xbook.vetmed.uni-muenchen.de/index_en.html. The animal bones are curated at Saarland University and available for research upon request. Photos of all the micro-remains recorded in the human dental calculus can be found in the Open Data LMU repository 10.5282/ubm/data.394.
