# Supplementary figures and images for: Reconstructing dietary practices at Tell Kamid el-Loz (Lebanon) during the Bronze and Iron Age III / Persian to Hellenistic periods using plant micro-remains from dental calculus and stable isotope analysis of bone collagen
Source: Archaeol Anthropol Sci. 2024 Jul 24;16(8):127. doi: 10.1007/s12520-024-02000-w (PMC11269519; doi:10.1007/s12520-024-02000-w)

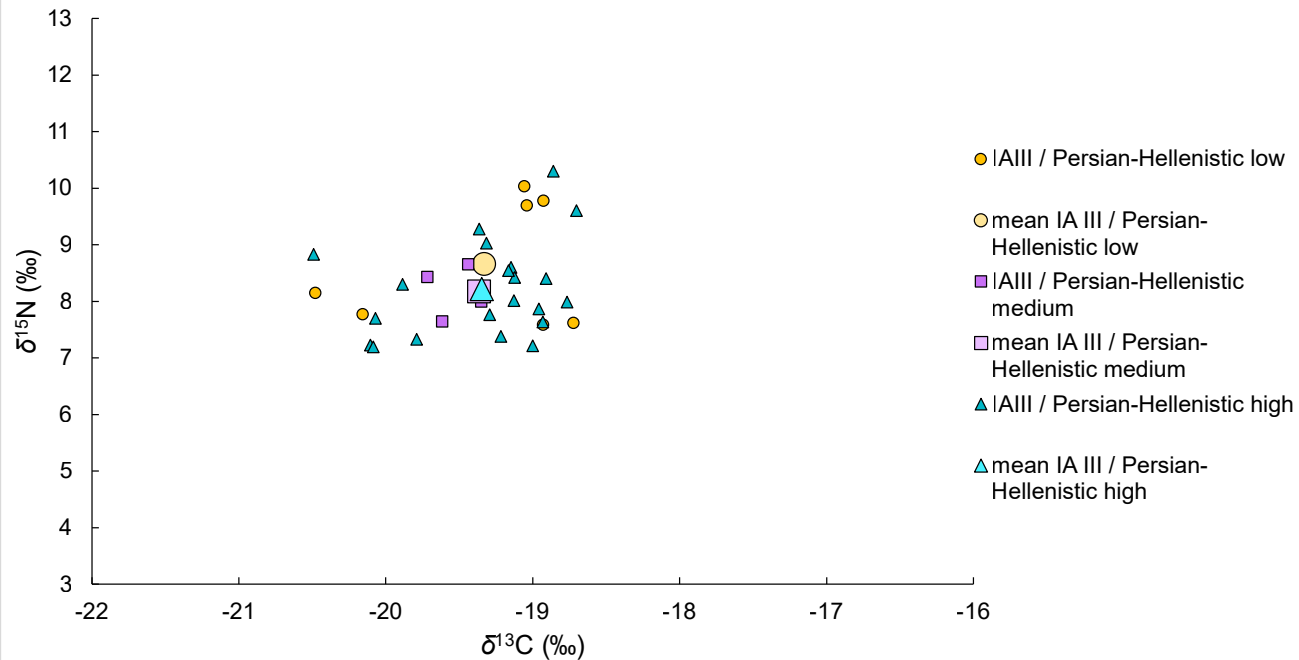

Supplement: Supplementary file 5 — Supplementary file5 (PDF 135 KB) [file 12520_2024_2000_MOESM5_ESM.pdf]
